# Text Mining and Drug Discovery Analysis: A Comprehensive Approach to Investigate Diabetes-Induced Osteoporosis

**DOI:** 10.7150/ijms.90829

**Published:** 2024-01-01

**Authors:** Chenfeng Wang, Yihe Hu, Feng Liang

**Affiliations:** Department of Orthopedic Surgery, The First Affiliated Hospital, College of Medicine, Zhejiang University, Hangzhou, Zhejiang 310030, China.

**Keywords:** Mining, Diabetes, Osteoporosis, Drug

## Abstract

**Purpose:** Osteoporosis (OP) and diabetes are prevalent diseases in orthopedic and endocrinology departments, with OP potentially arising as a complication of diabetes. However, the mechanism underlying diabetes-induced osteoporosis (DOP) remains enigmatic, and drug discovery in this domain is restricted, hindering research into the DOP's etiology and treatment. With the ultimate goal of preventing OP in diabetic patients, the objective of this study is to mine the genes and pathways linked to DOP using bioinformatics and databases.

**Method:** The present study employed text mining as the initial approach to retrieve genes commonly associated with diabetes and OP. Subsequently, functional annotation was conducted to investigate the roles and functionalities. In order to explore the interactions between proteins relevant to DOP, we constructed protein-protein interaction (PPI) networks. Furthermore, to obtain key genes and candidate drugs for DOP treatment, we conducted drug-gene interaction (DGI) analysis, complemented by a thorough examination of transcriptional factors (TFs)-miRNA co-regulation.

**Results:** The results through text mining revealed 110 genes that are commonly associated with both diabetes and OP. Subsequent enrichment analysis narrowed down the list to 95 symbols that were involved in PPI analysis. After DGI analysis, we identified 7 genes targeted by 11 drugs, which represent candidates for treating DOP.

**Conclusion:** This study unveils ANDECALIXIMAB, SILTUXIMAB, OLOKIZUMAB, SECUKINUMAB, and IXEKIZUMAB as promising potential drugs for DOP treatment, demonstrating the significance of utilizing text mining and pathway analysis to investigate disease mechanisms and explore existing therapeutic options.

## Introduction

Osteoporosis (OP) is a debilitating condition characterized by weakened bones and increased vulnerability to fractures. A complication arising from diabetes mellitus, OP manifests through reduced bone mass and microstructural changes in bone tissue 1. Despite a focus on disease regression, the underlying pathogenesis of diabetes-induced osteoporosis (DOP) remains a slow area of exploration. Additionally, addressing inefficient clinical treatment guidelines and limited drug discovery for DOP is of utmost importance.

The multifaceted realm of diabetes mellitus can be neatly divided into two categories, namely type 1 (T1DM) and type 2 diabetes mellitus (T2DM), each brandishing their distinct pathophysiological implications for the aggravated risk of fractures. It is fascinating to observe how both types display a conspicuous commonality in the form of decreased bone formation, osteoblast dysfunction, and low bone turnover 2. There is a possible association between diabetes and serum osteocalcin—notably, patients with either T1DM or T2DM have lower serum osteocalcin levels compared to non-diabetic individuals 3. Furthermore, osteocalcin levels demonstrate a negative correlation with glycosylated hemoglobin levels in T1DM patients 4. Only with a good understanding of the pathophysiology and pathogenesis of DOP can doctors be more effective in the treatment of these patients, such as developing targeted therapies and improving patient outcomes.

At present, there are very few available therapies for the prevention and treatment of DOP. Recent research suggests that physical activity can improve glucose intolerance and bone loss caused by diabetes, as well as promote skeletal development, possibly due to the effects of skeletal irisin 5. In addition, the interplay of choices takes center stage, with the selection of medication for patients ensnared in the clutches of both OP and diabetes evolving into a matter of paramount importance. The alluring first-line drug recommendation of bisphosphonates for OP therapy in diabetes-stricken patients whispers the promise of a brighter future 6. Scientific research verified that metformin has a positive effect on bone mineral density (BMD) in diabetics 7,8. However, the frowns return with the advisory not to embrace sulfonylurea drugs for individuals entangled in the web of bone mineral disorders and diabetes, for fear of the lurking menace of hypoglycemia 9, while thiazolidinediones are also not recommended due to their mechanism of action^10^. Therefore, it is crucial to prioritize research efforts in exploring potential pharmacological interventions given the increased demand from DOP patients.

With the rapid development of bioinformatics and drug discovery databases, new research ideas and strategies are given to the treatment of various diseases, which may be a potential way to explore the mechanism and therapeutic for the DOP. A therapeutic target database has been updated, which is facilitating the progress of drug discovery 11. Meanwhile, a simplified integrated database called TCMSID is exploited for mining candidate medicines from traditional Chinese medicine (TCM) 12. Additionally, a web-based knowledge graph database on synthetic lethality has also been created by researchers for the purpose of discovering new anticancer drugs 13. Therefore, making full use of existing bioinformatics technologies and drug databases may provide a shortcut to finding potential drugs for DOP.

The study aims to explore the pathomechanism and potential therapeutic options for DOP using text mining, functional annotation, protein network analysis, drug-gene interaction analysis, and miRNA-transcription factor (TF) coregulatory network construction, with the ultimate goal of informing clinical prevention and treatment for DOP.

## Method

### Text mining

Diabetes and OP-related gene sets were obtained through the GenCLiP3 website. In the GenCLiP3 database, using "All human genes" as the gene dataset, selecting "Search in MEDLINE" and using the default values in the journal filter, we searched for the keywords "diabetes" and "osteoporosis" respectively. Once the lists of associated genes are obtained, the intersection of the two sets of associated genes is utilized for functional enrichment analysis.

### Gene ontology (GO) and Kyoto Encyclopedia of Genes and Genomes (KEGG) analysis

GO and signaling pathway annotations for the genes corresponding to the intersection of OP and diabetes were carried out using the DAVID website. In addition, GO, which included biological processes, cellular components, and molecular functions, was a crucial method for achieving functional annotation. The key symbols that are closely related to the pathology of DOP were further mined using KEGG analysis. The false discovery rate (FDR) of the whole analysis should be less than 0.05.

### Protein-protein interaction (PPI) network

The common symbols were inputted into the STRING website among the high confidence (score 0.900) and the Cytoscape software to screen out hub genes by apps of Molecular Complex Detection (MCODE) and cytoHubba. The cutoff parameters were “degree cutoff=2”, “node score cutoff=0.2”, “k-core=2”, and “max depth=100”. And the cutoff parameters of MCODE were defaults, and that of cytoHubba were the top 10 genes.

### Drug-gene interaction (DGI)

Interacting genes between cytoHubba and MCODE apps were utilized for DGI analysis. The strict criteria are a DGI score transcending at a height of 3 and an unequivocal classification, the final genes were selected for the subsequent microRNA-transcription factor (TF) coregulatory network analysis.

### MiRNA-TFs regulatory network

The miRNAs and TFs associated with the final genes were investigated using the NetworkAnalyst platform.

## Result

### Attainment of common symbols

Following text mining, we identified 550 unique genes associated with diabetes and 172 unique genes related to OP. Among these, there were 110 common genes shared between diabetes and OP, forming the basis for further investigation.

### Functional annotations

The top 5 biological processes were (1) response to oxygen-containing compound (False discovery rate = 1.16E-48), (2) response to endogenous stimulus (False discovery rate = 8.68E-46), (3) response to organic substance (False discovery rate = 8.96E-45), (4) cellular response to chemical stimulus (False discovery rate = 2.68E-44), and (5) cellular response to organic substance (False discovery rate = 6.05E-43). Regarding cellular components, the top 5 were (1) extracellular space (False discovery rate = 1.01E-23), (2) extracellular region (False discovery rate = 1.03E-15), (3) extracellular region part (False discovery rate = 1.11E-14), (4) vesicle lumen (False discovery rate = 1.99E-08), and (5) cytoplasmic vesicle lumen (False discovery rate = 1.40E-07). As for the molecular function, (1) receptor binding (False discovery rate = 2.07E-26), (2) cytokine activity (False discovery rate = 6.54E-16), (3) hormone activity (False discovery rate = 4.46E-14), (4) identical protein binding (False discovery rate = 4.74E-13), and (5) cytokine receptor binding (False discovery rate = 3.00E-09) were the top 5.

**Figure 2:**
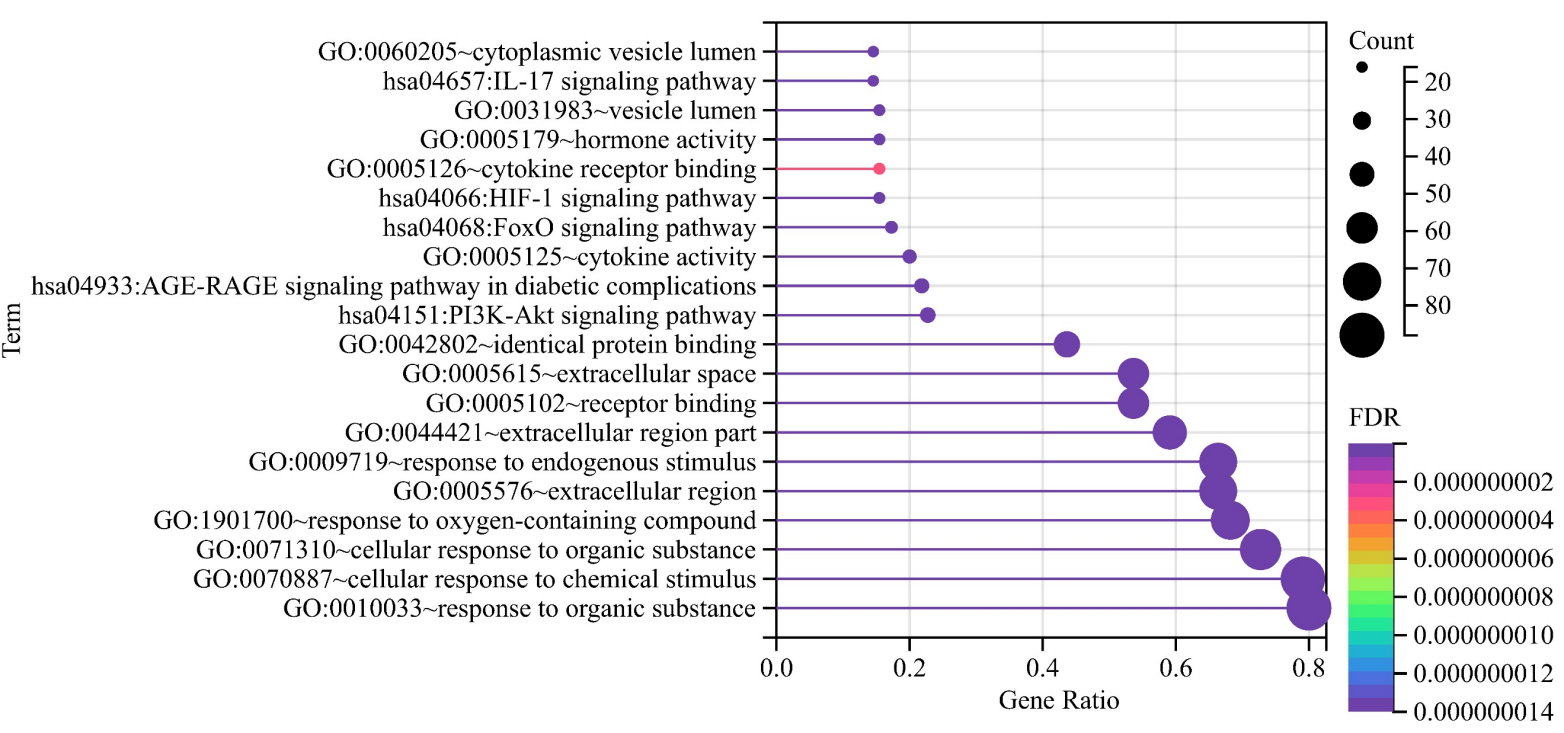
** Functional annotations.** With dots of varying size and color denoted the number of genes involved and their reliability, respectively.

KEGG pathway analyses revealed the involvement of (1) AGE-RAGE signaling pathway in diabetic complications (False discovery rate = 6.33E-22), (2) FoxO signaling pathway (False discovery rate = 1.55E-13), (3) HIF-1 signaling pathway (False discovery rate = 1.61E-12), (4) IL-17 signaling pathway (False discovery rate = 2.50E-12), and (5) PI3K-Akt signaling pathway (False discovery rate = 1.93E-12), hinting at potential mechanisms underlying DOP (Figure 2).

### PPI network and hub genes obtainment

**Figure 3:**
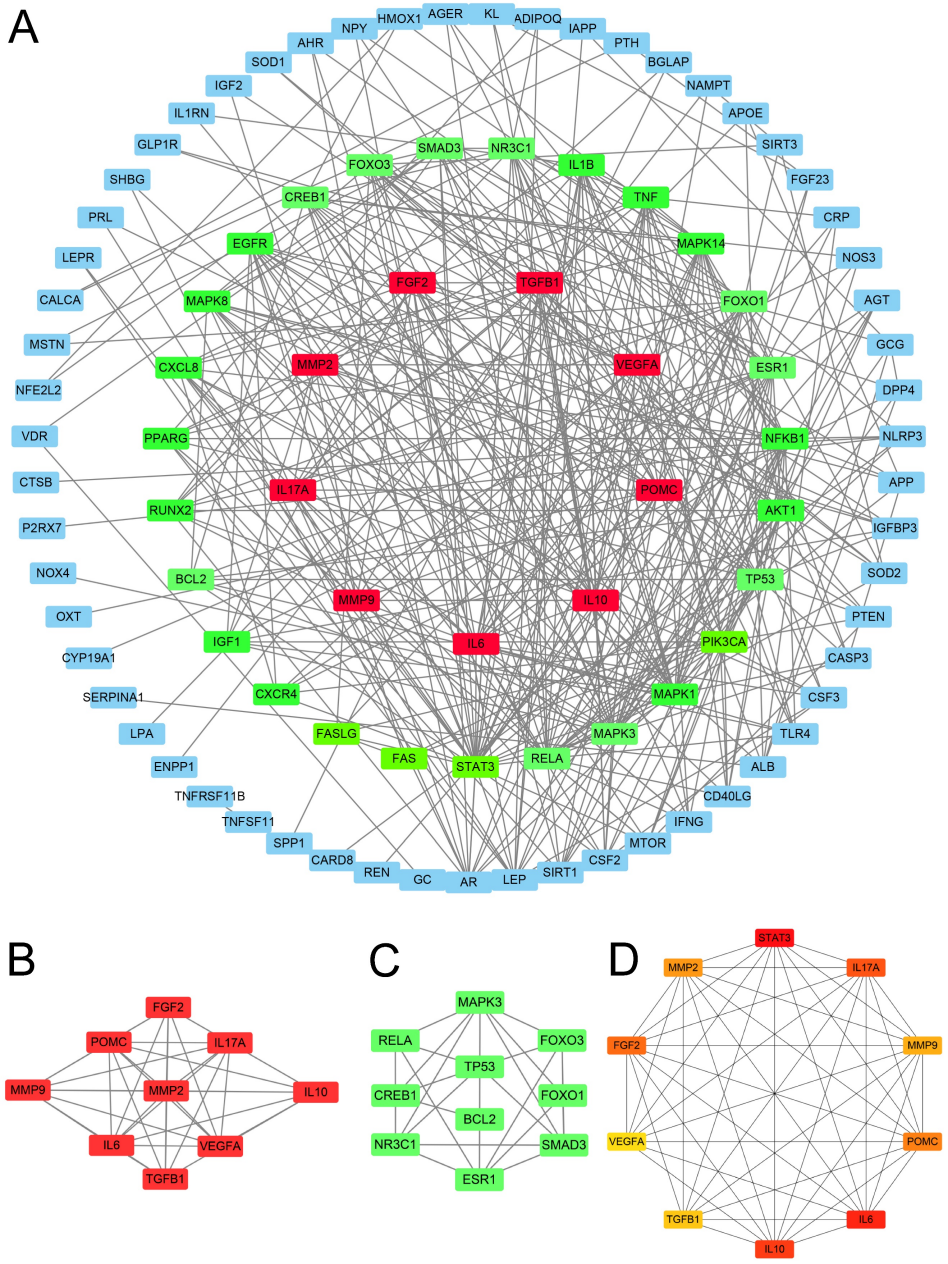
** Protein network analysis.** (A) The protein-protein interaction network was constructed using STRING. (B) Cluster 1: There are 35 edges and 9 nodes. (C) Cluster 2: There are 10 nodes and 21 edges. (D) CytoHubba analysis: The top 10 genes were analyzed.

The PPI network analysis yielded 67 genes with medium to high confidence interactions (Figure 3A). Subsequently, cluster analysis using MCODE identified 9 genes in the first module and 10 genes in the second module as hub genes (Figure 3B-C), while cytoHubba apps obtained the top 10 symbols (Figure 3D).

### Potential therapeutics

**Figure 4:**
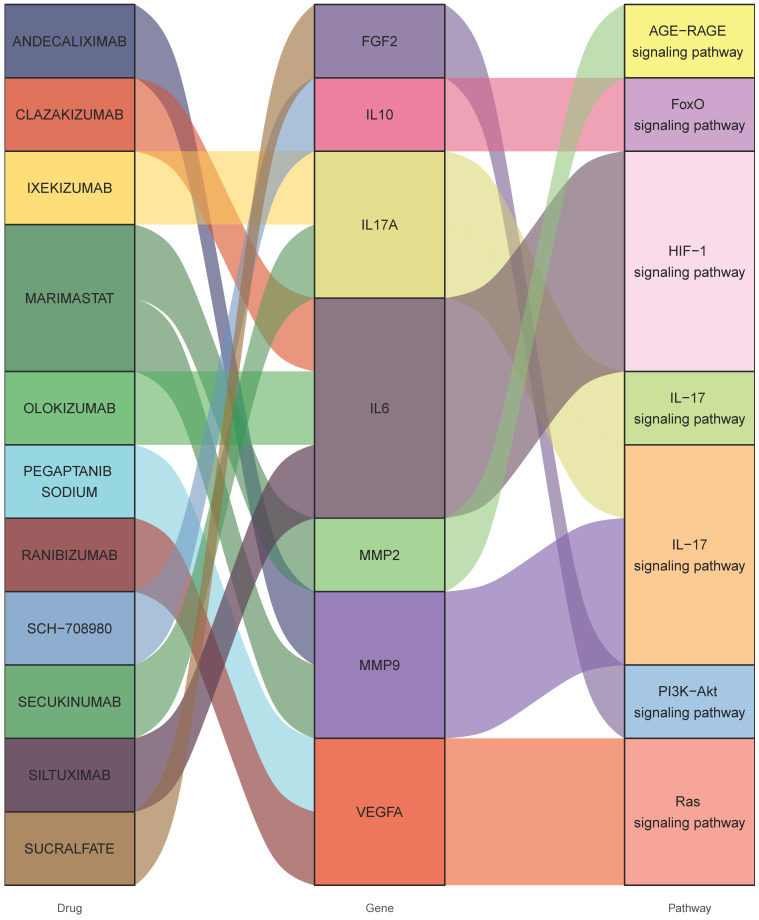
** Interactions between drug-gene-pathway.** The diagram provides insights into the interplay between 7 genes targeting 11 drugs and 6 pathways, providing an insightful visualization of the intricate relationships involved.

Consequently, 11 drugs (RANIBIZUMAB, PEGAPTANIB SODIUM, SUCRALFATE, SECUKINUMAB, IXEKIZUMAB, MARIMASTAT, SCH-708980, SILTUXIMAB, OLOKIZUMAB, CLAZAKIZUMAB, ANDECALIXIMAB) corresponding to 7 core genes (IL6-interleukin 6, VEGFA-vascular endothelial growth factor A, IL17A- interleukin 17 alpha, MMP2- matrix metallopeptidase 2, IL10- interleukin 10, FGF2- fibroblast growth factor 2, MMP9- matrix metallopeptidase 9) were found to affect DOP (Figure 4 and Table 1).

### MiRNA-TF coregulatory network

**Figure 5:**
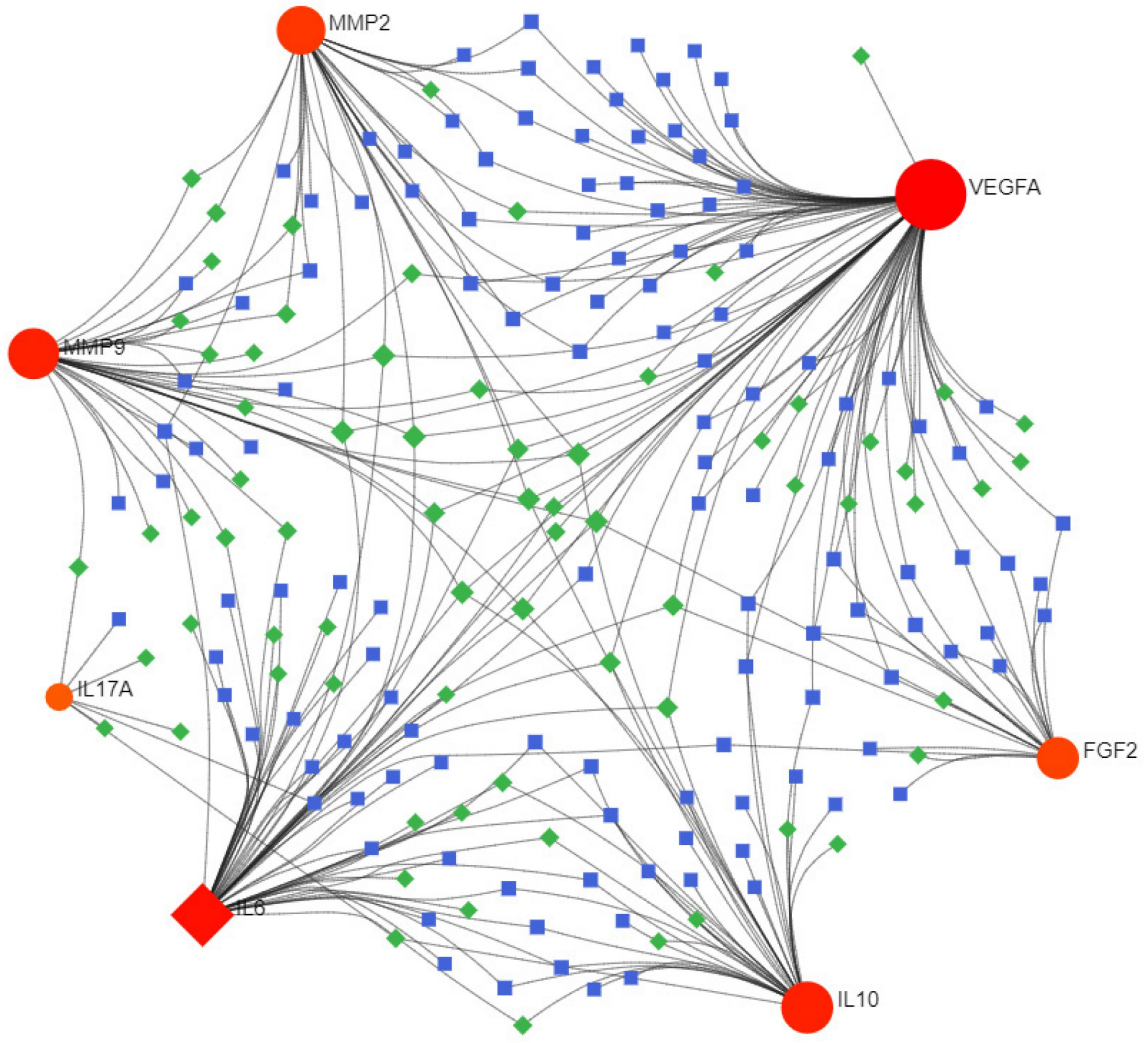
** TF-miRNA coregulatory network construction.** The TF-miRNA coregulatory network of the 7 hub genes is depicted, with red nodes signifying the hub genes, blue squares representing TFs, and green squares symbolizing miRNAs. This depiction offers a comprehensive understanding of the regulatory mechanisms at play.

The miRNA-TF coregulatory network analysis revealed five significant TFs, namely DPF2, ZBTB7A, ARID1B, IRF1, and CREM, and the top five miRNAs, including hsa-miR-107, hsa-miR-203a, hsa-miR-335, hsa-miR-15a, and hsa-miR-16, which might play important roles in the regulation of hub genes associated with DOP (Figure 5 and Table 2).

## Discussion

Diabetes can cause bone loss, which can lead to OP. The clinical prevention and treatment of DOP depend greatly on the definition of its mechanisms and therapeutic targets. The purpose of this study was to screen out key genes, identify targetable therapeutic agents and explore the underlying pathogenesis through multiple bioinformatics methods.

Consequently, 7 hub genes correlated to 11 drugs and 6 pathways were filtrated. FGF2, produced by epithelial and mesenchymal cells, plays a crucial role in skeletal development and the formation of osteoblasts 14,15. However, its expression decreases in a high-glucose environment, leading to osteoblast proliferation disorder 16. IL10, renowned for its potent immune and inflammatory suppression properties, is thought to inhibit bone resorption and reduce bone loss 17, but some studies have proved that IL10 is significantly increasing in DM patients 18,19, which also just goes to show that DOP development and its regulatory mechanisms are quite complex. Moving on to IL17A, a proinflammatory cytokine, it can indirectly stimulate bone resorption by RANKL (receptor activator of NF-κB ligand)^20^,^21^. Besides, diabetes induces cells to secrete IL17A, which in turn leads to a variety of complications 22.

Additionally, this investigation delved into IL6, an inflammatory factor known not only to encourage osteoclastogenesis, resulting in excessive osteoclastic activity and osteolysis 23, but also involves in the pathophysiology of diabetes 24. HIF-1 controls VEGFA, an efficient therapeutic target for OP, to encourage bone formation. Reduced bone formation could result from OP if its expression is reduced 25. When it comes to MMP2 and MMP9, they are playing a key role in bone physiology. On the one hand, the expression of MMP-2 and MMP-9 are negatively correlated with bone mineral density and considered as the essential biomarkers of OP 26. On the other hand, they can hold potential as biomarkers useful in predicting the risk and progression of T2DM 27.

As mentioned above, the expressions of IL17A, MMP2, IL10 and IL6 are up-regulated with diabetes that promote the progression of OP, whereas the expressions of FGF2 and VEGFA are down-regulated. Therefore, this study only addresses inhibitors of up-regulated genes. Based on the DGI scores, the top 5 potential drugs are ANDECALIXIMAB (MMP9 inhibitor, 10.3), SILTUXIMAB (IL6 inhibitor, 9.89), OLOKIZUMAB (IL6 inhibitor, 9.89), SECUKINUMAB (IL17A inhibitor, 7.73) and IXEKIZUMAB (IL17A inhibitor, 7.73). ANDECALIXIMAB is mainly applied for gastric cancer and ulcerative colitis treatment 28,29, and it is currently in the clinical trial stage. SILTUXIMAB and OLOKIZUMAB, both as IL6 inhibitors, are now respectively being studied for Castleman disease 30 and rheumatoid arthritis 31. SECUKINUMAB and IXEKIZUMAB are used in clinical trials for the therapy of plaque psoriasis and psoriatic arthritis with a remarkable therapeutic effect 32-34. Despite not being currently used for DOP treatment, targeting these key genes may hold the potential to prevent the onset and progression of the disease.

It is worth noting that miRNA and TF may play a big part in the appearance and regulation of DOP. For example, a study shows that circulating miR-107 facilitates osteogenesis and serves as a potential diagnostic biomarker and therapeutic target for OP 35, but miR-107 is dysregulated in diabetes 36,37. IRF1, as a transcription factor (TF), promotes osteogenic differentiation 38 and is also involved in the progress of diabetes 39. Therefore, miRNA and TF may also be an important breakthrough in the fight against DOP.

DOP, characterized by metabolic bone loss, involves the endocrine system as a crucial mediator. Advanced glycation end products (AGEs) interact with the receptor for AGEs (RAGE) and may significantly impact bone cell metabolism and function 40. The AGE-RAGE pathway emerged as a critical player in diabetic complications, including diabetic osteopathy, potentially contributing to reduced bone density, suppressed bone turnover markers, and impaired bone quality in diabetic patients 41,42. AGEs play a crucial role in both OP and diabetes. They not only promote osteoclastogenesis by increasing RANKL expression but also negatively impact osteoblasts 43,44. Another important pathway involved in bone and immune cell regulation is the IL-17 signaling pathway, where elevated plasma IL-17 levels are closely linked to BMD 45. The PI3K-Akt signaling pathway is essential for inhibiting OP through promoting bone formation 46, whereas in the context of diabetes, the pathway becomes impaired, exacerbating the pathogenic cascade. The aforementioned key genes are intricately involved in the pathogenesis of both diabetes and OP, suggesting that post-diabetic up- or down-regulation of genes significantly contributes to the occurrence and progression of OP via complex pathways, including the PI3K-Akt signaling pathway and AGE-RAGE pathway. Consequently, perturbations in the delicate balance between osteogenesis and osteoclasts lead to the manifestation of OP.

Of particular concern is the potential for these interacting genes to engender a vicious cycle, thereby exacerbating the severity of both OP and diabetes. As such, early interventions in diabetes patients through targeted medical therapy present an advantageous approach to inhibit the development of OP. This approach encompasses the control of the internal environmental disturbances inherent in diabetes to promote physiological stability, as well as the timely implementation of interventions to postpone the progression of OP and diabetes, ultimately disrupting the deleterious cycle.

Unfortunately, this study is not without limitations. The dynamic nature of the utilized database necessitates periodic updates to maintain congruence with ongoing developments and to ensure replicability. Moreover, the subjectivity involved in the criteria for core genes, such as the parameter choices for application analysis (MCODE analysis and PPI construction) and DGI score selection, warrants consideration and further refinement. To bolster the credibility of this investigation, experimental verification remains imperative.

## Conclusion

In conclusion, the present study successfully identified a total of 110 intersecting genes that are implicated in both diabetes and OP. The implementation of functional annotation has revealed a potentially innovative avenue for understanding the mechanisms underlying DOP. Consequently, a refined selection comprising seven genes (MMP2, MMP9, IL17A, IL10, FGF2, IL6 and VEGFA) and eleven pharmacological agents has emerged, exhibiting promise in delivering therapeutic effects aimed at mitigating the occurrence of OP in individuals afflicted with diabetes.

## Supplementary Material

Supplementary information.

## Figures and Tables

**Figure 1:**
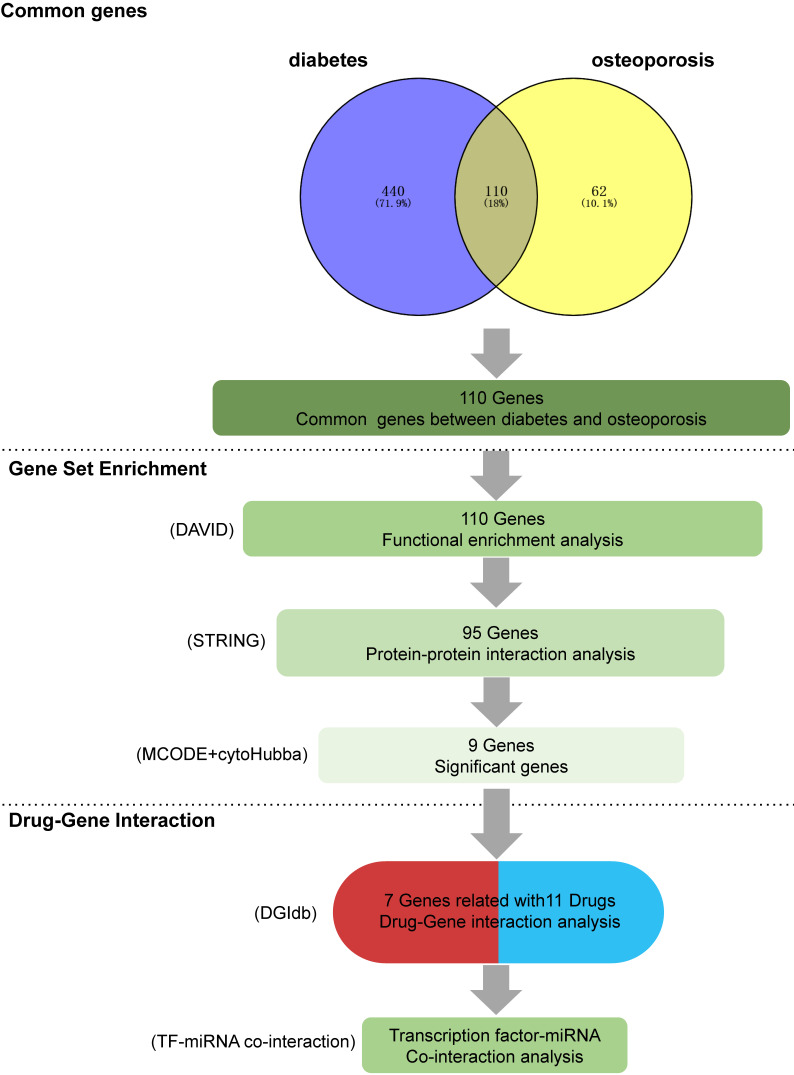
** A comprehensive summary of the overall results obtained from data mining.** (1) Intersecting genes: the process of obtaining intersecting genes involved the utilization of specific search terms, 'diabetes' and 'osteoporosis' in GENCLIP3. This yielded 550 genes for diabetes and 172 genes for OP, ultimately leading to the identification of 110 common genes shared between the two conditions (2) Functional annotations: gene functional enrichment analysis was performed using the DAVID tool. Subsequently, 9 hub symbols were identified using STRING and Cytoscape software. (3) Drug-gene interaction analysis: 11 potential medicines were acquired by DGIdb. For the final TF-miRNA co-interaction analysis, seven genes were chosen.

**Table 1:** Candidate drugs for diabetes-induced osteoporosis

Number	Name	Symbol	Type	Score	PMID
1	RANIBIZUMAB	VEGFA	Inhibitor	8.54	18046235
2	PEGAPTANIB SODIUM	VEGFA	Antibody, Agonist	3.25	23953100
3	SUCRALFATE	FGF2	Agonist	5.89	7948825
4	SECUKINUMAB	IL17A	Antibody, Agonist	7.73	25354738
5	IXEKIZUMAB	IL17A	Antibody, Agonist	7.73	None
6	MARIMASTAT	MMP2	Inhibitor	3.31	15748894
7	SCH-708980	IL10	Inhibitor	3.64	None
8	SILTUXIMAB	IL6	Inhibitor	9.89	8823310
9	OLOKIZUMAB	IL6	Inhibitor	9.89	24641941
10	CLAZAKIZUMAB	IL6	Inhibitor	7.42	None
11	ANDECALIXIMAB	MMP9	Inhibitor	10.3	None
12	MARIMASTAT	MMP9	Inhibitor	3.68	17234180

Each drug-gene interaction was rigorously assessed to ensure that the putative drug exerted the anticipated effect on the specific medical condition, whereby the screening criterion necessitated an interacting score exceeding 3. Subsequently, the provenance of the information was diligently monitored to validate the report's veracity and appraise associated metadata. Drugs that effectively targeted the candidate genes via suitable interactions were subsequently aggregated in the conclusive inventory. * The total number of Pubmed and database references determines the score.

**Table 2:** Summary of TFs and miRNA

TFs	Symbols	Count
DPF2	IL6, IL17A	2
ZBTB7A	IL6, IL17A	2
ARID1B	IL6, FGF2	2
IRF1	IL6, FGF2	2
CREM	IL6, FGF2	2
hsa-miR-107	IL6, IL17A, FGF2	3
hsa-miR-203a	IL6, IL17A, FGF2	3
hsa-miR-335	IL6, FGF2, VEGFA	3
hsa-miR-15a	IL6, IL17A	2
hsa-miR-16	IL6, IL17A	2

## Abbreviations

OP: osteoporosis
DOP: diabetes-induced osteoporosis
PPI: protein-protein interaction
DGI: drug-gene interaction
TF: transcriptional factors
T1DM: type 1 diabetes mellitus
T2DM: type 2 diabetes mellitus
BMD: bone mineral density
GO: Gene ontology
KEGG: Kyoto Encyclopedia of Genes and Genomes
FDR: false discovery rate
RANKL: receptor activator of NF-κB ligand
AGEs: advanced glycation end products
