# Supplementary material for: Text Mining and Drug Discovery Analysis: A Comprehensive Approach to Investigate Diabetes-Induced Osteoporosis
Source: Int J Med Sci. 2024 Jan 1;21(3):464–73. doi: 10.7150/ijms.90829 (PMC10797669; doi:10.7150/ijms.90829)
Supplement: Supplementary file 1 — Supplementary information. [file ijmsv21p0464s1.pdf]

110 common elements in "diabetes" and "osteoporosis":
LPA
GCG
ALB
GLP1R
DPP4
CRP
ADIPOQ
LEP
IAPP
REN
AVP
PPARG
IGF1
TNF
VEGFA
IL6
AGT
AKT1
EGR3
AGER
APOE
NOS3
SIRT1
IL10
PTH
MTOR
NLRP3
SOD1
NFE2L2
IFNG
TGFB1
SHBG
VDR
IL1B
BGLAP
EGFR
NAMPT
FNDC5
TLR4
POMC
LEPR
PRL
TNFRSF11B
MAPK1
MAPK8
FOXO1
NFKB1
HMOX1
IL17A
APP
MMP9
CASP3
HMGCR
CALCA
NPY
IGFBP3
BCL2

TP53
RARRES2
CARD8
OXT
MTHFR
MAPK14
SPP1
FGF23
CD40LG
STAT3
IGF2
SOD2
FAS
PIK3CA
ENPP1
MMP2
SOST
IL1RN
NOX4
KL
MAPK3
CXCL8
GC
FASLG
CTSB
NR3C1
TNFSF11
FGF2
SIRT3
MSTN
SERPINA1
CXCR4
ALPP
PTEN
CSF3
MIR21
ALDH2
CREB1
SMAD3
AR
CSF2
ESR1
MALAT1
CYP19A1
METTL3
RELA
AHR
GNRH1
FOXO3
BMP7
DKK1
P2RX7
RUNX2
